# Trial of labour or elective repeat caesarean delivery:are women making an informed decision at Kenyatta national hospital?

**DOI:** 10.1186/s12884-017-1440-3

**Published:** 2017-08-15

**Authors:** Phocas Biraboneye S, Omondi Ogutu, Jos van Roosmalen, Samson Wanjala, Kizito Lubano, John Kinuthia

**Affiliations:** 1 Department of Obstetrics and Gynaecology, Chateau-Thierry Hospital Center, Nairobi, France; 2 Department of Obstetrics and Gynaecology, University of Nairobi, Nairobi, Kenya; 3 Athena Institute, VU University Amsterdam, Amsterdam, The Netherlands; 4 Department of Obstetrics and Gynaecology, University of Nairobi, Nairobi, Kenya; 5 Kenya Medical Research Institute, Nairobi, Kenya; 6 Department of research and programs, Kenyatta National Hospital, Nairobi, Kenya

## Abstract

**Background:**

Trial of labour is a safe option for most women after one previous caesarean delivery. However, the proportion of women attempting trial of labour after previous caesarean delivery (TOLAC) has been declining in many countries. In addition, women with prior caesarean delivery appear to know little regarding their mode of delivery and healthcare providers’ recommendations. The doctors’ preferences exert a strong influence on patient’s decision whether or not to pursue TOLAC. In Kenya, it is unclear whether women who opt for trial of labour after caesarean delivery (TOLAC) or elective repeat caesarean delivery (ERCD) do that based on clear understanding of risks and benefits of both modes of delivery. This study aimed at determining whether patients with one previous caesarean delivery make an informed decision on preferred mode of delivery following their interactions with doctors.

**Methods:**

A cross-sectional descriptive study was carried out on 202 pregnant women with one previous caesarean delivery at Kenyatta National Hospital (KNH) antenatal clinic. Data was collected from both the patients’ records and women were interviewed using a structured questionnaire.

**Results:**

Out of 202 women with mean age of 30.2 years 136 (67.2%) chose Elective Repeat Caesarean Delivery (ERCD), while 66 (32.8%) opted for TOLAC. Only 61/202 (30.6%; 95% C.I: 24.4 to 37.6%) made informed decisions. Few women (65: 32.2%) knew that the chance of successful TOLAC was high (60-80%) and 97 (48%) were not aware of the chances for a successful TOLAC. More than half of the women (109: 53.9%) were unaware of the risk of uterine rupture after one previous delivery and only few patients (64: 31.7%) knew that the risk of uterine rupture in TOLAC is low (< 1%). The majority of the women (112: 55.4%) did not know that the indications for previous caesarean delivery are an important factor in determining the chance of a successful Vaginal Birth after Caesarean Delivery (VBAC). For 47(23.3%) of the women, there was no documented indication for the previous caesarean delivery. The women’s mode of delivery was significantly associated with the preference of the counseling doctor (*p* < 0.001) and their qualification (*p* = 0.020). Only 23 (11.4%) women signed the consent form for ERCD while none of the women for TOLAC signed any consent form.

**Conclusions:**

There was an overall lack of information on both modes of delivery while doctor’s preferences affected women’s decisions. Only just under one third of the women made an informed decision. There is a need to develop clear standard protocols and checklists for information to be disseminated to doctors and all patients with previous caesarean deliveries in subsequent pregnancies in Kenya.

**Electronic supplementary material:**

The online version of this article (doi:10.1186/s12884-017-1440-3) contains supplementary material, which is available to authorized users.

## Background

A successful trial of labour culminating in vaginal birth, a failed trial resulting in an emergency repeat caesarean delivery, or an elective repeat caesarean delivery are the three possible outcomes for the woman who has had a prior caesarean section. Planning the mode of delivery should be addressed early during prenatal care, and can begin even pre-conceptionally. With either approach, women who have undergone a prior caesarean delivery are at risk for serious maternal and perinatal complications and should be counselled about these risks [1].

In the USA, Federal Acts and regulations as well as professional guidelines clearly demonstrate that every pregnant woman has the right to base her maternity care decisions on accurate, up-to-date, comprehensible information [2]. Informed consent is "the willing acceptance of a medical intervention by a patient after adequate disclosure by the physician of the nature of the intervention and its alternatives with its risks and benefits " [3, 4]. It is thus more than just signing a consent form.

The decision for elective repeat caesarean delivery or trial of labour should be made by the woman in consultation with her provider. Both clinicians and patients desire individualized information about the chance of successful TOLAC and the balance between the risk of maternal or foetal morbidity if TOLAC is unsuccessful and the risk of maternal and foetal morbidity with ERCD [5]. Women in the USA report that their healthcare providers’ recommendations and preferences exert a strong influence on their decision whether or not to pursue TOLAC [6].

This information is also important on a population level, especially in the setting of rising caesarean delivery rates, as selection of candidates who are most likely to deliver vaginally after a previous operation can minimize the costs of ERCD and failed TOLAC [5]. In fact, improving patient education may not affect the increasing section rate; it would, however, empower women to make a well informed, educated decision [7].

The aim of this study was to determine whether patients with one previous caesarean delivery were making an informed decision about their preferred mode of delivery at “Kenyatta National Hospital”.

## Methods

### Study sites

The study was conducted in the Antenatal Clinic at KNH, which is the national referral hospital located in Nairobi. It is also the main teaching hospital for the College of Health Sciences, University of Nairobi. The hospital caters for patients from Nairobi and its neighbourhoods as well as referrals from other hospitals in the country.

### Study design

This was a cross-sectional descriptive study that targeted pregnant women with one previous caesarean delivery attending ANC at KNH for delivery from September 2013 to January 2014. On average approximately 50 women with one previous caesarean delivery attend ANC per month.

### Data collection

Consenting women completed structured questionnaires to assess their demographic characteristics, awareness about risks and benefits of both modes of delivery, doctors’ preferences, knowledge about complications following prior caesarean delivery as well as indications of previous caesarean delivery, and knowledge about the mode of delivery in the current pregnancy. Data abstraction was also done from patients’ records through a structured questionnaire to assess parity, level of provider, family planning goals, and relevant clinical examination including estimated foetal weight, obstetrical ultrasound data, timing on decision of mode of delivery and any other medical or obstetric complication (Additional file 1).

### Statistical analysis

Data was entered into a micro-computer through Epidata designed database and exported to STATA 11.0 and Statistical Package for Social Science (SPSS-20) for analysis. Comparative analysis focused on patient knowledge, baseline characteristics, obstetric characteristics and antenatal care provided, and chosen mode of delivery. *P* < 0.05 was considered statistically significant.

### Ethics

Approval was obtained from the KNH/UoN Ethics and Research committee before carrying out the study. Informed consent of women was obtained before participating in the study. Antenatal care was provided to all women regardless of whether they consented or declined to participate in the study. The records were coded and anonymised. The information collected remained confidential and was only used for the purpose of the study. No incentives were given to the study subjects.

## Results

### Socio-demographic characteristics

A total of 202 women participated in the study with a mean age of 30.2 years. The highest proportion of women (77; 38.1%) was in the group between 30 and 34 years (Table 1). Most women were married (94%). Many women (*n* = 92; 45.6%) had secondary level of education and 46% of the participants were self-employed. The majority of women with one previous caesarean delivery were para one (75.7%). The outcome of the previous caesarean delivery was a live birth for 82.1%. About 15.8% (*n* = 32) reported medical or obstetric complications following the previous caesarean delivery, with 39.5% among them reporting early neonatal death. The majority of the women (89.6%) opted for no contraceptive choice following the current delivery, with only 5.9% of the women preferring bilateral tubal ligation. Before attending ANC –KNH, 47% of the patients preferred ERCD as mode of delivery while 42.6% of the patients preferred TOLAC (Table 2).

**Table 1:** Socio-demographic characteristics of the study participants

Parameters	*N* (%)
Woman’s age (years)	
20-24	25 (12.4%)
25-29	64 (31.7%)
30-34	77 (38.1%)
> 35	36 (17.8%)
Educational level	
Primary	38 (18.8%)
Secondary	92 (45.6%)
University	72 (35.6%)
Marital Status	
Married	190 (94.0%)
Single	10 (5.0%)
Separated	2 (1.0%)
Occupation	
Employed	49 (24.3%)
Business /self employed	92 (45.5%)
Student	2 (1%)
Housewive	59 (29.2%)

**Table 2:** Obstetric characteristics of the study participants

Parameters	*N* (%)
Woman’s parity	
1	153 (75.7%)
2-3	45 (22.3%)
> 3	4 (2%)
Outcome of previous caesarean delivery	
live infant	166 (82.1%)
Live infant in distress	4 (2%)
Premature baby	1 (0.5%)
Still birth	11 (5.4%)
Neonatal death	10 (5%)
Infant death	6 (3%)
Not documented	4 (2%)
Medical or obstetrical complications after the previous c/s according to the participant’s interview (*n* = 32)	
Bleeding (PPH)	3 (7.9%)
Eclampsia	8 (21.1%)
Maternal infections	8 (21.1%)
Death of the baby(neonatal death)	15 (39.5%)
Burst abdomen	3 (7.9%)
Post partum psychosis	1 (2.6%)
Women’s family planning method choice following the current delivery	
Bilateral Tubal Ligation	12 (5.9%)
Natural	1 (0.5%)
Pills	6 (3%)
Injections	2 (1%)
Not yet decided	181 (89.6%)
Women’s preferred mode of delivery before attending KNH	
Trial of labour	86 (42.6%)
Elective repeat c/s	95 (47.0%)
Not sure	17 (8.4%)
Any	4 (2.0%)

### Antenatal clinical assessment

Non reassuring foetal heart rate (NRFS) was the most common indication for the previous caesarean delivery documented at 27.2% (Table 3). However, for 23.3% of the women there was no documented indication. The medical or obstetric complications postpartum were not documented for the majority (98%). During the time of decision of mode of delivery, the estimated foetal birth weight was not documented in 90.6% and only 11.4% of the consent forms for ERCD was signed and documented in the file, while patients for TOLAC did not sign any consent form.

**Table 3:** Antenatal clinical assessment of women with one previous caesarean delivery attending KNH ANC

Parameters	*N* (%)
Medical or obstetrical complication documented during ANC	
Preeclampsia	8 (4%)
Diabetes	2 (1%)
Anemia	1 (0.5%)
HIV-positive	7 (3.4%)
Placenta praevia	2 (1%)
Malpresentation	5 (2.5%)
Fibroids	5 (2.5%)
No complication documented/indicated	172 (85.1%)
Documented reasons for the previous caesarean delivery	
CPD/big baby	31 (15.3%)
Malpresentation	15 (7.4%)
APH	4 (2%)
Failed induction	18 (8.9%)
Prolonged labour	32 (15.8%)
Non reassuring foetal distress	55 (27.2%)
Not documented	47 (23.3%)
Medical or obstetrical complication postpartum documented after the previous caesarean delivery	
Infections	2 (1%)
Preeclampsia	2 (1%)
Not documented	198 (98%)
Estimated foetal birth weight (Kg) clinically or by U/S documented at the time of the decision of mode of delivery	
< 3.5	15 (7.4%)
≥ 3.5	4 (2%)
Not documented	183 (90.6%)
Consent form signed and documented in the file at the time of decision for mode of delivery	
Yes (Women admitted for ERCD from ANC)	23 (11.4%)
Booked in elective diary	92 (45.8%)
Not documented	86 (42.8%)

### Participants’ knowledge on risks and benefits of both modes of delivery.

On complications of surgery, most women knew little about complications of anaesthesia (8.3%), injury to organs, severe haemorrhage, hysterectomy and even maternal death (1.1%) as associated risks of repeat caesarean delivery.

Only 32.2% of the women knew that the overall chances of success of TOLAC is >50%. Regarding the risks of uterine scar rupture, more than half of the women (53.9%) did not know about this risk, while only 31.7% knew that the risk is very low (< 1%), but increasing with the number of repeat caesarean deliveries (Table 4). More than half of the women (55.4%) did not know that the reasons for previous caesarean delivery are an important factor in determining the chance of successful TOLAC.

**Table 4:** Knowledge on risks and benefits of both modes of delivery amongst one previous caesarean delivery women attending KNH ANC

Parameters	*N* (%)
Women’s knowledge on risks associated with repeat C/S than TOLAC (*n* = 163)	
Increased blood loss	124 (28.4%)
High risk of infection	106 (24.3%)
Complication of anaesthesia	36 (8.3%)
Uterine scar rupture in case of big baby	10 (2.3%)
Injury to organs	5 (1.1%)
Recovery is longer	140 (32.2%)
Limb numbness	15 (3.4%)
Women’s knowledge on risks associated with TOLAC than ERCD (*n* = 155)	
Uterine scar rupture resulting in emergency C/S	54 (20.8%)
Failed trial of labour	114 (44%)
Uterine rupture is > with VBAC than repeat C/S	81 (31.3%)
Increased blood loss	5 (1.9%)
Increased risk of infection	2 (0.8%)
Foetal death	3 (1.2%)
Women’s knowledge regarding overall chances of success of TOLAC	
Very high (>50%)	65 (32.2%)
Very low (<25%)	40 (19.8%)
Don’t know	97 (48%)
Women’s knowledge regarding the risks of uterine scar rupture	
Very low (<1%) but increased with number of c/s	64 (31.7%)
Very high (>50%)	29 (14.4%)
Don’t know	109 (53.9%)
Women’s knowledge on recovery from vaginal delivery against repeat C/S	
Same	1 (0.5%)
Longer for repeat C/S	166 (82.2%)
Longer for vaginal delivery	2 (1%)
I don’t know	33 (16.3%)
Reasons for previous c/s as important factor in determining the chance of successful TOLAC according to the participants	
Yes	60 (29.7%)
No	30 (14.9%)
Don’t know	112 (55.4%)

The majority of the participants (70.3%) had attended ANC at KNH more than three times and in 62.4% of the cases women were informed on ERCD as the only mode of delivery.

Few women knew that a classical scar (7 out of 116) and unavailability of 24 h theatre and skilled providers (1/116) are reasons to consider recommending ERCD (Table 5). The majority of doctors at ANC- KNH were senior house officers (174: 86.1%), while consultants were represented at 28 (13.9%).

**Table 5:** Number of antenatal visits and information provided to women with one previous caesarean delivery attending KNH ANC

Parameters	*N* (%)
Number of ANC visits at the time of recruitment	
< 3	60 (29.7%)
≥ 3	142 (70.3%)
Women informed on available options of mode of delivery	
Elective repeat c/s	126 (62.4%)
Trial of labour	61 (30.2%)
None	15 (7.4%)
Women counselled on reasons for ERCD against TOLAC (*n* = 116)	
Big baby (>3.5 kg)	21 (18.1%)
Classical scar	7 (6%)
Small pelvis	15 (12.9%)
Unavailability of 24 h theatre/blood transfusion/skilled doctors and anaesthetists	1 (0.9%)
Mal-presentation/breech presentation	11 (9.5%)
Bad obstetric history	3 (2.6%)
Choice of BTL	2 (1.7%)
Placenta praevia	21 (18.1%)
Don’t know	35 (30.2%)
Women counselled at discharge after previous C/S on reasons ERCD is recommended in subsequent pregnancy (*n* = 19)	
Small pelvis	13 (68.5%)
Classical uterine incision	2 (10.5%)
High risk of uterine rupture if any VBAC	2 (10.5%)
BTL would also be offered	2 (10.5%)
Counselling doctor preferred mode of delivery (perception of the participant)	
None	44 (21.8%)
Elective repeat caesarean section	107 (53%)
Trial of labour	51 (25.2%)
Level of provider at ANC KNH	
Senior house officer	174 (86.1%)
Consultant obstetrician	28 (13.9%)

The decision made on mode of delivery during ANC was ERCD in 136 (67.2%) women and trial of labour in 66 (32.8%). After counselling during ANC a significant reduction in the choice of TOLAC was observed from 42.6% to 32.8% with an increase from 47% to 67.2% of ERCD (Table 6).

**Table 6:** Decision or choice of TOLAC and ERCD before attending KNH ANC and after counseling

Before KNH-ANC visit (TOLAC)	After ANC visit (TOLAC)		
	Yes	No	Total
Yes	58	28	86 (42.6%)
No	8	108	116
Total	66 (32.8%)	136	
Before KNH-ANC visit (ERCD)	After ANC visit (ERCD)		
	Yes	No	
Yes	91	4	95 (47%)
No	45	62	107
Total	136 (67.2%)	66	

In Table 7, the minimum proposed criteria for informed decision on mode of delivery at KNH are given. On average, it was reported that 61 (30.2%; 95% CI: 24 to 37%) of the women were making informed decisions.

**Table 7:** Minimum criteria for a woman with one previous caesarean delivery to make an informed decision on mode of delivery at KNH

Criteria	*N* (%)
Women informed on mode of delivery (available options) (*n* = 202)	
TOLAC	61 (30.2%)
ERCD	126 (62.4%)
Both	0
Women’s knowledge on factors influencing mode of delivery (*n* = 116)	
Classical scar as a reason for ERCD	7 (6%)
Small pelvis as a reason for ERCD	15 (12.9%)
Big baby as a reason for ERCD	21 (18.1%)
Current medical or obstetric complications as a reason for ERCD	30 (25.9%)
Adequacy of facility for delivery (A 24 h-theater…)	1 (0.9%)
Women’s knowledge on success and risk of uterine rupture in TOLAC (*n* = 202)	
Patient knowledge on overall chance of TOLAC success	65 (32.2%)
Patient knowledge on risk of uterine rapture in TOLAC	64 (31.7%)
Reasons of previous caesarean as factor of success for TOLAC	60 (29.7%)
Women’s knowledge on risks of ERCD over TOLAC (*n* = 163)	
Increased blood loss	124 (76.1%)
High risk of infection	106 (65.0%)
Complication of anaesthesia	36 (22.1%)
Injury to organ	5 (3.1%)
Recovery is longer	140 (85.9%)
Consent form signed and documented in the patient file (*n* = 202)	23 (11.4%)

There was no association with preferred mode of delivery in terms of women’s age (*p* = 0.654), level of education (*p* = 0.224), marital status (*p* = 0.419) number of ANC visits (*p* = 0.574), patient’s parity (*p* = 0.286, and patient occupation (*p* = 0.795).The following correlates were, however, significantly associated with the preferred mode of delivery: counselling doctor preferred mode of delivery (*p* < 0.001), women preferred mode of delivery before attending KNH (*p* < 0.001) and level of care provider (*p* = 0.02) (Table 8).

**Table 8:** Antenatal and obstetric correlates associated with the preferred mode of delivery amongst one previous caesarean delivery women attending KNH ANC

Parameters	ERCD *n* = 136	TOLAC *n* = 66	*P*-value
Women’s parity			
1	104 (76.5%)	49 (74.2%)	0.286
2-3	28 (20.6%)	17 (25.8%)	
> 3	4 (2.9%)	0	
Number of ANC visits at the time of recruitment			
< 3	35 (26.3%)	19 (30.2%)	0.574
≥ 3	98 (73.7%)	44 (69.8%)	
Counselling doctor preferred mode of delivery			
None	29 (21.3%)	15 (22.7%)	0.001
Repeat caesarean section	107 (78.7%)	0	
Vaginal delivery	0	51 (77.3%)	
Women’s preferred mode of delivery before attending KNH			
Trial of labour	28 (20.6%)	58 (87.9%)	0.001
Elective repeat c/s	91 (66.9%)	4 (6.1%)	
Not sure	14 (10.3%)	3 (4.5%)	
Any	3 (2.2%)	1 (1.5%)	
Level of provider at ANC KNH			
Senior house officer	123 (90.4%)	51 (78.5%)	0.020
Consultant	13 (9.6%)	14 (21.5%)	

No association with preferred mode of delivery was found in terms of estimated foetal birth weight at the time of decision (*p* = 0.372) and knowledge on risks associated with repeat CS as compared to TOLAC (*p* = 0.482). However, knowledge of women regarding overall chances of success of TOLAC (*p* < 0.001), risk of uterine rupture in TOLAC (*p* < 0.001) and women’s knowledge on recovery from vaginal delivery against repeat caesarean delivery (*p* < 0.001) were significantly associated with the preferred mode of delivery (Table 9).

**Table 9:** Women’s knowledge correlates associated with the preferred mode of delivery amongst one previous caesarean delivery patients attending KNH ANC

Parameters	ERCD *n* = 136	TOLAC *n* = 66	*P*-value
Women’s knowledge regarding the risks of uterine scar rupture			
Very low (<1%) but increased with number of c/s	17 (26.6%)	47 (73.4%)	0.001
Very high (>50%)	27 (93.1%)	2 (6.9%)	
Don’t know	92 (84.4%)	17 (15.6%)	
Women’s knowledge regarding overall chances of success of TOLAC			
Very high (>50%)	12 (8.8%)	53 (80.3%)	0.001
Very low (<25%)	40 (29.4%)	0	
Don’t know	84 (61.8%)	13 (19.7%)	
Women’s knowledge on recovery from vaginal delivery against repeat C/S			
Same	-	1 (1.5%)	0.001
Longer for repeat C/S	103 (75.7%)	63 (95.5%)	
Longer for a vaginal delivery	2 (1.5%)	0	
I don’t know	31 (22.8%)	2 (3%)	
Estimated foetal birth weight in Kg clinically or by U/S at the time of the decision of mode of delivery			
< 3.5	10 (7.4%)	5 (7.6%)	0.372
≥ 3.5	4 (2.9%)	-	
Not documented	122 (89.7%)	61 (92.4%)	
Women’s awareness on risks associated with repeat C/S than TOLAC			
Increased blood loss	76 (26.6%)	48 (32%)	0.482
High risk of infection	65 (22.7%)	41 (27.3%)	
Complication of anaesthesia	26 (9.1%)	10 (6.8%)	
Rupture in case of big baby	8 (2.8%)	2 (1.3%)	
Injury to organs	3 (1%)	2 (1.3%)	
Recovery is longer	96 (33.6%)	44 (29.3%)	
Limb numbness	12 (4.2%)	3 (2%)	

## Discussion

The study findings show that few women chose TOLAC and even fewer were making an informed decision. Most women preferred repeat caesarean delivery before attending ANC at KNH and this was significantly associated with the patient’s choice after ANC counseling. Equally, women’s mode of delivery was significantly linked with the preference of the counselling doctor and their qualification. Women appear to know little about their mode of delivery.

However, this study did not establish an association between preferred mode of delivery and women’s demographic characteristics (age, educational level, marital status, occupation, parity) and number of antenatal visits.

A small number of women chose TOLAC probably because of inadequate information and influence of the counselling doctor from ANC. A recent study published by Sarah and collaborators has demonstrated the same findings [6]. The majority of the women did not make an informed decision at KNH and this was probably because of lack of adequate information provided in ANC or largely due to poor women counselling. This study proposes minimum criteria for an informed decision in a woman with one previous caesarean delivery attending KNH. No similar studies involving clear criteria to determine whether they are making informed decisions have been performed.

However, worldwide the practice of obstetrics and gynecology has always faced special ethical questions in the implementation of informed consent [2]. More studies need to be done for a generalization of the proposed criteria. Most of the women did not know the overall chances of success (60-80%) and the risks of uterine rupture (<1%) with TOLAC. These findings are different from the study done by Sarah and collaborators in USA [6] and the difference is probably due to Sarah’s questionnaire administered to the women at admission and not immediately during the antenatal visits.

There was a significant correlation with women’s preferred mode of delivery before attending ANC KNH and chosen mode of delivery after ANC counseling (*p* < 0.001). This was probably due to alternative sources of information and poor antenatal education regarding both modes of delivery. Most factors influencing mode of delivery in women not counselled on mode of delivery during antenatal visits were identified as friend advice and internet information. No similar study has shown the alternative source of information besides health providers counselling on decision making. In this study majority of the senior house officers were offering repeat caesarean delivery due to probably less experience and lack of standard guidelines in management of a woman with one previous caesarean delivery.

According to Wells (2010), physicians that did not offer VBAC were also more likely to be practicing <10 years, have been involved in a law suit related to caesarean delivery or have experienced uterine rupture with maternal or foetal complications [8]. Most inexperienced providers tend to have bias towards informed consent of patients with one previous caesarean delivery. In addition, some patients having limited understanding of pregnancy and child birth would be easily influenced by the most senior doctors. However some challenges associated with informed consent are to be remembered: information that may be considered necessary or desirable in formally educated urban populations may be of little relevance in less formally educated or rural populations, or vice versa. Also, in some cultures it might not be customary to provide certain forms of information, such as describing uncertainty about the effectiveness of the treatment, or information about possible alternative treatments.

Some of the providers therefore apply the dictum of Craigin: “Once a caesarean, always a caesarean” [9]. Because informed consent laws and principles do not specify the amount of information that must be disclosed, physicians might find it useful to know what they have to disclose.

In literature, advanced maternal age, single marital status and less than 12 years education are associated with a reduced likelihood of successful TOLAC [10]. In addition, this study found a limited documentation in patient’s files. Proper documentation is a useful tool in informed consent. Without proper documentation a patient may not get adequate and relevant information. Researchers have also shown that the patients may not accurately remember all the facts disclosed in a discussion [11, 12] In practice, there is no consent form for TOLAC patients available in Kenya. Few patients signed the consent form for ERCD while none did for TOLAC. Consent forms should be available at the moment of decision to prove that the patient was given information, understood and consented.

Some of the main limitations of this study are the small sample size and lack of standardization in women counselling. However, this study has two major strengths: it is original and proposes minimum criteria for an informed decision in women with prior caesarean delivery attending KNH. Data was obtained from an institution offering VBAC and the population was not only more educated but also more urbanized and middle class than the average across the country. The results may therefore represent a better informed population suggesting wider knowledge gaps throughout the country. This study should be considered as a preliminary evaluation of current practice and patterns. It is intended therefore, to provoke further interest in the subject of making informed decisions by women with previous caesarean deliveries.

## Conclusions and recommendations

### Conclusions

There is overall lack of information on both modes of delivery while doctor’s preference affects patient’s decision. There is lack of documentation in patient’s files as well on minimum proposed criteria for informed decision at KNH.

### Recommendations

Develop a standard protocol and policy for management of women with a previous caesarean section attending KNH. Need for training of providers using such a protocol on the importance of documentation.

## Additional file

Additional file 1: Questionnaire. (DOCX 31 kb)

## Abbreviation

ANC: Antenatal Clinic
C/S: Cesarean Section
ERC: Ethics Research Committee
ERCD: Elective Repeat Cesarean Delivery
KNH: Kenyatta National Hospital
MD: Medical Doctor
MMed: Master in Medicine
RCD: Repeat Cesarean Delivery
RCOG: Royal College of Obstetricians and Gynecologists
SPSS: Statistical Package for Social Science
TOL: Trial of Labor
TOLAC: Trial of Labor after Cesarean Delivery
USA: United States of America
VBAC: Vaginal Birth after Cesarean Delivery

## References

[1] Wells EC, Cunningham GF. Choosing the route of vaginal delivery. www.uptodate.com. Accessed 4 Aug 2014.

[2] ACOG (2004). Updates of ethical decision making in obstetrics and gynaecology in ethics obstetrics and gynaecology.

[3] Paul S, Appelbaum M (2007). Assessment of patients ‘competence to consent to treatment. N Engl J Med.

[4] Michael S, Anthony B, Jillann F (2012). The guide to informed decision-making in healthcare.

[5] Torri DM (2014). Use of calculators for predicting successful of labour after caesarean delivery.

[6] Sarah N, Matalon S, Rosenn B, et al. Trial of labour versus repeat: are patients making an informed decision? Am J Obstet Gynecol. 2012;207:204. 10.1016/j.ajog.2012.06.057 22939727

[7] The American Academy of Pediatrics and the American College of Obstetricians and Gynecologists (2007). Guidelines for Perinatal Care. J Contin Educ Heal Prof.

[8] Wells C (2010). Vaginal birth after delivery: views from a private practitioner. Semin Perinatol.

[9] Edwin C (1916). Conservatism in obstetrics. New York Medical Journal.

[10] Srinivas SK, Stamilio DM, Samuel MD (2007). Vaginal birth after caesarean delivery: does maternal age affect safety and success?. Paediatr Perinat Epidemiol.

[11] Robinson G, Meray A (1976). Informed consent: recall by patients tested postoperatively. Ann Thorac Surg.

[12] Herz DA, Looman JE, Lewis SK (1992). Informed consent: is it a myth. Neurosurgery.

