# Supplementary material for: Trial of labour or elective repeat caesarean delivery:are women making an informed decision at Kenyatta national hospital?
Source: BMC Pregnancy Childbirth. 2017 Aug 15;17:260. doi: 10.1186/s12884-017-1440-3 (PMC5558758; doi:10.1186/s12884-017-1440-3)
Supplement: Additional file 1: — Questionnaire. (DOCX 31 kb) [file 12884_2017_1440_MOESM1_ESM.docx]

## Questionnaire

**Part I: To interview the patient by investigator or Assistant investigator**

Identification

1. Patient Number: …………….
2. Patient’s age: ……………….
3. Indicate your highest education level attained:
4. University
5. High school(secondary)
6. Primary
7. None above
8. Indicate the number of antenatal clinic attendance (visits): ………………..
9. Indicate your marital status:
10. married
11. single
12. separated
13. divorced
14. Indicate your occupation:
15. Employed
16. business
17. student
18. housewife

**Please answer these questions based on the counseling you have received in ANC from medical professionals and your own general knowledge:**

1. Before coming to antenatal clinic (clinic 18), what was your preferred mode of delivery?
2. Trial labor
3. Repeat C/S
4. Not sure
5. Any
6. None
7. When you came to ANC, did your doctor inform you about the different options of mode of delivery?
8. No
9. If yes, which one?
10. Trial of labor
11. Repeat cesarean delivery
12. Both above
13. In antenatal clinic, did your doctor advice you about the risks and benefits of both mode of delivery?
14. Yes
15. If no your decision on mode of delivery was based on what? Tick all applied.
    - 1. A friend advice
      2. Internet information
      3. Cost of mode of delivery
      4. Others. Specify
16. What is the decision made on mode of delivery during ANC?
17. Trial of labor
18. Repeat C/S
19. Not yet
20. Do you feel your doctor preferred one method of delivery over another?
21. my doctor did not have a preference
22. my doctor preferred that I have a repeat cesarean section
23. my doctor preferred that I try for a vaginal delivery
24. Are you aware about the risks associated with cesarean delivery?
25. No
26. If yes, which one of the following complications is more associated with repeat C/S than a VBAC (vaginal delivery after C/S)? Tick all applied
27. Increased blood loss
28. High risk of infection
29. Complication of anesthesia
30. Rupture in case of big baby
31. Injury to organs (in the mother)
32. Recovery is longer
33. Others. Specify
34. Don’t know
35. Are you aware about the risks associated with trial of labor (VBAC)?
36. No
37. If yes, which of the following risks are greater for a woman having a VBAC compared to a repeat C/S? Tick all applied
    1. Risk of uterine rupture resulting in emergency C/S
    2. Risk of failed trial of labor
    3. Risk of uterine rupture is higher with VBAC than repeat C/S
    4. More blood loss
    5. More risk of infection
    6. Others. Specify………………………………………..
    7. Don’t know
38. If you were to try for a trial of vaginal labor, your overall chances of success are:
39. Very high (>50%)
40. Very low (<25%)
41. Don’t know
42. If you try for a vaginal delivery (VBAC), the risk that your uterus will rupture (opening of the uterine scar) is:
43. Very low (<1%), but increases each time you have another cesarean section
44. Very high (>50%)
45. Don’t know
46. Your recovery from a successful vaginal delivery versus a repeat cesarean section is:
47. The same
48. Longer for a repeat cesarean section
49. Longer for a vaginal delivery
50. I do not know
51. Choose the correct statement about Repeat cesarean section and VBAC. Tick all applied
52. VBAC presents higher risk than ERCD
53. ERCD presents higher risk than VBAC
54. VBAC does not present any benefits
55. ERCD does not present any benefit
56. Don’t know
57. Did your doctor inform you about any reasons why you should better deliver by ERCD than trial of labor (VBAC)?
58. No
59. If yes, which one? Tick all applied
60. Big baby (> 3.5kg)
61. Type of uterine incision (classical scar)
62. small pelvis
63. availability of theater, blood transfusion, experienced doctors
64. Others. Specify
65. Don’t know
66. Why did you have the previous cesarean delivery?
67. Small pelvis
68. Big baby
69. Fetal distress
70. Transverse lie
71. Bleeding (APH)
72. Others. Specify……………………..
73. Don’t know
74. Have you had any complications after the previous cesarean delivery?
75. No
76. If yes which one? Tick all applied
    - 1. Bleeding (PPH)
      2. Convulsions or increased blood pressure
      3. Maternal infections
      4. Death of the baby
      5. Others. Specify……………………………
77. Was ERCD recommended to you as mode of delivery in subsequent pregnancy at discharge?
78. No
79. If yes, what reasons were you given?
80. ERCD because of small pelvis
81. ERCD because classical uterine incision
82. ERCD because of high risk of uterine rupture if any trial of labor (VBAC)
83. ERCD because BTL will also offered.
84. Others. Specify
85. Have you ever had a successful vaginal delivery following your cesarean section?
86. Yes
87. No
88. The reason for your previous cesarean section is an important factor in determining your chances of a successful vaginal delivery:
89. Yes
90. No
91. I do not know
92. How are you satisﬁed with the language communication of your doctor during ANC discussion?
93. Satisﬁed
94. Not satisﬁed
95. How satisﬁed are you with your previous cesarean section experience?
96. Satisﬁed
97. Not satisﬁed
98. In ANC, have you been given time to ask questions for clarification:
99. Yes
100. No

**Part II: To be filled by investigator from Antenatal cards or patient’s file**

1. Patient number:
2. Level of provider:
3. Clinical officer or Midwife
4. General practitioner
5. Senior house officer
6. Consultant
7. Parity of the patient: …………………..
8. Family planning method choice following delivery:
9. BTL
10. DIU transcesarean section
11. Other, and specify…………………………
12. Not yet decided
13. Indication of the previous CS:
14. Contracted pelvis or cephalopelvic disproportion
15. Transverse lie
16. APH
17. Other and which one? ........................................
18. Not indicated in the patient file
19. Estimated fetal weight birth in Kg clinically or by ultrasound documented at the time of the decision of mode of delivery:
20. < 3.5Kg
21. ≥ 3.5Kg
22. Not indicated
23. Medical or obstetrical complication noted during ANC:
24. Preeclampsia
25. Cardiac disease
26. Diabetes
27. Other and which one? …………...........................
28. Not indicated / documented
29. Medical or obstetrical complication postpartum noted after the previous CS delivery: Tick all applied
30. Infections
31. Hemorrhages
32. Anesthetic complications
33. Longer recovery
34. Others. Specify…
35. Not indicated
36. Outcome of the first delivery:
37. Well live infant
38. Live infant in distress
39. Premature baby
40. Still birth
41. Others. Specify…
42. Not indicated
43. Patient had a vaginal delivery after the first cesarean delivery:
44. Yes
45. No
46. Not indicated
47. Patient induced in the first pregnancy:
48. If yes with what?
49. No
50. Not indicated
51. Patient mode of delivery in the first pregnancy:
52. Elective cesarean delivery
53. Emergency cesarean delivery
54. Obstetrical ultrasound done on this pregnancy:
55. No
56. If Yes. The latest at which gestation………....
57. Not indicated
58. Decision on the mode of delivery indicated in the file on this pregnancy:
59. No
60. Yes. If yes which one and at which gestational age (GA)?
61. ERCD and at what GA……………(in weeks of amenorrhea)
62. Trial of labor (TOLAC) and at what GA……..(in weeks of amenorrhea)
63. Is the estimated fetal weight birth (clinically or by ultrasound) during this ANC indicated at the decision of mode of delivery?
64. Yes. If yes, which one (in Kg)?
65. No
66. Not indicated
67. Consent form signed and dated in the file:
68. Yes
69. Patient booked in elective diary
70. Not indicated
71. Booked ERCD is to be done at which GA (in weeks)? .......................................
